# A case study exploring the impact of JCI standards implementation on staff productivity and motivation at the laboratory and blood bank

**DOI:** 10.1002/hsr2.497

**Published:** 2022-01-19

**Authors:** Rima Ramzi Abou Tarieh, Ramez Zayyat, Rania Nazir Naoufal, Hanady Rafic Samaha

**Affiliations:** ^1^ Faculty of Science Lebanese University Beirut Lebanon; ^2^ Laboratory and Blood Bank Department Saint Georges Hospital University Medical Center (SGHUMC) Beirut Lebanon

**Keywords:** change management, joint commission international, laboratory and blood Bank, motivation, productivity, quality management

## Abstract

**Background and Aims:**

Quality of care has transformed to become an essential element of healthcare service delivery, which caused decision makers in Health Care Organisations (HCOs) to seek methods to evaluate the level of care provided. The field of accreditation is under massive development, especially in healthcare organizations. Joint Commission International (JCI) accreditation is one of the accreditation bodies that require a lot of documentation and quality improvement to guarantee proper standard application. The process of accreditation is known to be demanding and requires staff involvement to guarantee successful implementation.

**Methods:**

This study focuses on the impact of JCI standards implementation on staff productivity and motivation in a 350‐bed hospital. An interpretive approach was used to collect empirical data by interpreting the population's behavior, which is represented in this study by the questionnaire. The study is considered an emergent study that identified, explained, illustrated, and developed a model of staff motivation. In this type of study, the process of inquiry, supporting arguments, and questions of interest is developed after the launching of the study and during the process of data collection. Thus, the researcher relied on the social constructive paradigm, whereby the grounded theory (GT) is used to build the research model of staff motivation during the process of accreditation.

**Results:**

By studying the Turn Around Time (TAT) performance indicator, the study showed that TAT of tests decreased by 3% after JCI standards implementation, which was reflected at two different laboratory sections. The trainings conducted throughout the process of standards implementation also resulted in enhancing the quality of samples, which was demonstrated by the decrease in the percentage of rejected samples.

**Conclusion:**

Staff productivity increases when JCI standards are implemented. However, staff motivation is dependent on their involvement with management decisions and the smooth transition through change management, which ensures staff retention and therefore increase productivity.

## 1 INTRODUCTION

Quality of care has transformed to become an essential element of healthcare service delivery, which caused decision makers in Health Care Organisations (HCOs) to seek methods to evaluate the level of care provided. Accreditation is a formal process in which an authorized body evaluates and assesses an organization's compliance with a set of pre‐designed performance standards.^1^ Healthcare leaders and top management bodies in HCOs consider accreditation as being an effective approach to improving the quality of care provided. Moreover, the process of accreditation is considered exhaustive and requires top management commitment as well as proper resource management, which includes staff involvement throughout the complete progression. This costly process is under a dilemma of whether the application of these pre‐set standards is effective and how the accreditation, certification impacts the human talents at the HCO, mainly the staff of the Laboratory and Blood Bank.

The Joint Commission is known to be the “oldest health care accrediting body in the world”.^2^ Its standards aid HCOs toward improving the quality of care, as well as it responds to the extended needs of health care organizations. Health care organizations tend to improve their performance through accreditation by means of meeting the pre‐set standards through policies, procedures, and protocols, whereby those are made in compliance with the targeted accreditation bodies recommendations. The process of policy development, protocol formulation, and implementation require proper resource allocation, as well as staff motivation and commitment.

### 1.1 The impact of accreditation on the quality of diagnostic services provided by the laboratory

The definition of quality in health care varies with respect to the dimensions of the stakeholders, which includes and is not limited to patients, healthcare workers and third‐party payers. To establish a simple and accurate definition of quality, we will refer to quality as a balance between what the organization plans and what the customer expects, which will be linked to how the organization delivers the services and how the customer perceives it. This will add sustainability and consistency into the loop, which can be achieved through standardization. The health care sector is controlled by several factors such as price, competitive advantage also affected by the market structure, publicity and mainly the quality of care provided.^3^ Laboratories have developed three main pillars; quality, clinical effectiveness, and cost effectiveness. The main priority is the quality of services provided since it directly impacts the two others. Laboratories tend to improve quality through decreasing diagnostic errors, reducing turnaround times of tests performed and standardization of all laboratory procedures through policies, procedures, and protocols. Laboratories aim toward accreditation due to several factors, an example of which is competency demonstration which acts as a competitive advantage, improving quality of service, and satisfying managerial interests. Also, accreditation can be considered as a stimulant of continuous quality improvement, which keeps the quality system alive at the HCO. However, the accreditation process is considered a time and resource consuming activity, thus the dilemma lies between to accredit or not to accredit a Clinical Laboratory.

### 1.2 The development of JCI accreditation in the clinical laboratory and blood bank

In 2010, the Joint Commission International (JCI) developed the Accreditation standard for clinical laboratories (second edition) which is derived from its general quality standards that provides hospital accreditation. The significance of JCI accreditation lies within the development of quality management practices in hospital laboratories. The JCI accreditation combines a standard with its corresponding guideline, which defines the intent of each standard and the unique measurable elements of each requirement. Laboratory and Blood Bank standards fall beneath the Assessment of Patients (AOP) chapter, which includes all the diagnostic services provided by the hospital. Safety requirements are the most significant in this chapter, however, it is the only document that does not cover all the total Quality System Elements (QSE); whereby the information management aspect is not tackled. JCI standards do not give the Blood Bank and Transfusion services specific standards and guidelines, however, the process of organization and quality assurance is similar to that applied in the Clinical Laboratory. The importance of JCI accreditation in the Clinical Laboratory and Blood Bank lies within the standardization of processes and the importance of documentation to guarantee the tracking of services, minimizing errors and securing continuous quality and performance improvements.

### 1.3 Change management through the JCI accreditation process

Change management is known to be the ability to redirect the processes, structures, and capabilities of an organization in response to external or internal alterations.^4^ Implementing changes in health care is difficult since it requires the collaboration of people from different orientations. The process of JCI accreditation is subject to constant pressure to improve processes, methods and technologies at the Laboratory and Blood Bank. This will include and will not be limited to changes at the workforce level, methodology and automation, sectional organization, policies, procedures, and programs as well as excessive documentation. Quality control measures are subject to enormous resistance since they are culture dependent. The importance of sectional organization lies within optimization of Laboratory performance through reducing the Turn Around Time (TAT) of samples which improves the patient care in the organization. This is achieved by the unification of test procedure and maximizing automation. The main adjustment required by JCI standards is policy and procedure development, which ensures standardization. Managerial efforts in standardization have been successful in quality improvement, mainly in repetitive production and administrative processes.^5^ Quality improvement through standardization frequently meets resistance, which delays the process of implementation and requires excessive managerial effort. Staff resistance to the changes enforced by JCI standards will interrupt the process of implementation and therefore accreditation. To ensure the proper implementation and to secure accreditation, Laboratory and Blood Bank management have to manage change in a way that sets a comfortable environment for staff. Subsequently, process standardization impacts different performance measures, and remarkable quality.^5^ This implies that while developing policies and procedures, staff involvement will be crucial to guarantee compliance and thus appliance. “People are not always receptive to change”.^4^ Thus, it is the management's role to design the implementation of change. The process of implementation includes knowledge and education, methods, and techniques to facilitate the understanding and utilization of change. Employees resist change due to a variety of reasons, most importantly lack of understanding of the reasons to change an accustomed mechanism. Communication is the most fundamental tool to promote change, thus it is important to present the winning situation and confirm transparency. If the change will not under any terms secure a winning situation, the management should have the courage to withdraw without being vulnerable to pressure.

### 1.4 Design and methodology

This study aimed at assessing the impact of JCI standards implementation on the Clinical Laboratory and Blood bank staff's productivity and motivation. It was conducted in one of the largest hospitals in Beirut. It relies on the key performance indicators to assess the staff productivity before JCI standards implementation and throughout the process of implementation and development. An employee engagement survey was distributed and considered different variables present among the staff, including age, educational background, and working experience. A qualitative emergent approach was used, which was selected as the best approach to be primarily used in this study based on the interpretive epistemological paradigm. In an interpretive approach, qualitative researchers are known to study things in their “natural settings”, by interpreting a phenomenon in terms of the way people bring meaning to them.^6^ An interpretive approach was used to collect empirical data by interpreting the population's behavior, which is represented in this study by the questionnaire. The qualitative approach relies on the observation and examination of the targeted population which allows the researcher to derive the variables present.^7^ The study is considered an emergent study that identified, explained, illustrated, and developed a model of staff motivation. In this type of study, the process of inquiry, supporting arguments, and questions of interest is developed after the launching of the study and during the process of data collection. The use of this design is due to the lack of situational control and inadequate understanding of the actual problem to be studied. Thus, the researcher relied on the social constructive paradigm, whereby the grounded theory (GT) is used to build the research model of staff motivation during the process of accreditation. The main purpose of GT is to integrate the qualitative and quantitative research methodologies in terms of developing action processes.^8^

A questionnaire was built to assess staff motivation during standard implementation. Analysis of the questionnaire was made on Statistical Package for the Social Sciences (SPSS). Demographic descriptive data was extracted from this questionnaire. The aim of this study was to assess staff productivity and motivation during the process of JCI standards implementation, thus the study focused on staff experience, motivation, satisfaction, and productivity. For further analysis, the relationship between these variables was also studied. In order to assess the significance of the relationship between the above variables, a two‐tailed chi‐square test of significance was conducted on SPSS. Since the sample is relatively small (less than 30), Fisher exact test is used to determine the correlations between the qualitative variables.

The population included all Laboratory and Blood Bank staff in the hospital. The questionnaire was built to be applied at the hospital, where the processes of JCI standards implementation are initiated. The study was granted the International Review Board (IRB) approval prior its launching. An informed consent, in English and Arabic was attached before each questionnaire to verify the voluntary participation as well as the ethical collection of data and therefore analysis.

To study the staff's performance during the process of JCI standards implementation, which is reflected by the Clinical Laboratory's performance, Key Performance Indicators (KPIs), were derived from the Laboratory department, targeting the turnaround time of STAT (Short Turn Around Time) tests defined by the Laboratory. KPIs are established after thorough analysis of accreditation documents, performance appraisals, and process completion. STAT tests are defined as tests to be reported in a short turnaround time, usually below 1 h. The Clinical Laboratory categorizes Electrolytes, Troponin, Creatinine, CBC, and Urine analysis STAT tests. Data were extracted from the Laboratory Information System (LIS), and compared as prior to the installation of the pre‐analytical system and the formation of the core laboratory as well as post development and initialization of the previously described upgrades along the months of September 2019, December 2019, April 2020, May 2020, June 2020, and July 2020.

Since data are collected and interpreted from a sample of staff members of a hospital in Lebanon in order to emphasize the meaning of their experience with quality improvement and JCI standards implementation, the grounded theory approach was used to build a new form of management model in accordance with the ADKAR change management model. The research strategy also included a meta‐analysis of the theoretical and empirical examination of self‐administered questionnaires, the TAT reports of the STAT tests throughout the process of core lab implementation as well as specimen rejection criteria as per JCI standards recommendations. Quantitative analysis is also included in this study in the form of descriptive statistics and simple correlations. Meta‐analysis was conducted in different context in order to determine an overall trend. It was initially developed in the field of education by Glass (1976) in order to blend quantitative research in the field of psychology.^9^ In this study, meta‐analysis was presented through the thorough review of qualitative research approaches in management, followed by accreditation report analysis which highlights of the changes imposed by JCI standards and the management's efforts in implementing change as well as the staff's response to development. As a result, a change management model is developed to secure a healthy working environment, thus increasing productivity and motivation.

## 2 RESULTS AND DISCUSSION

**FIGURE 1:**
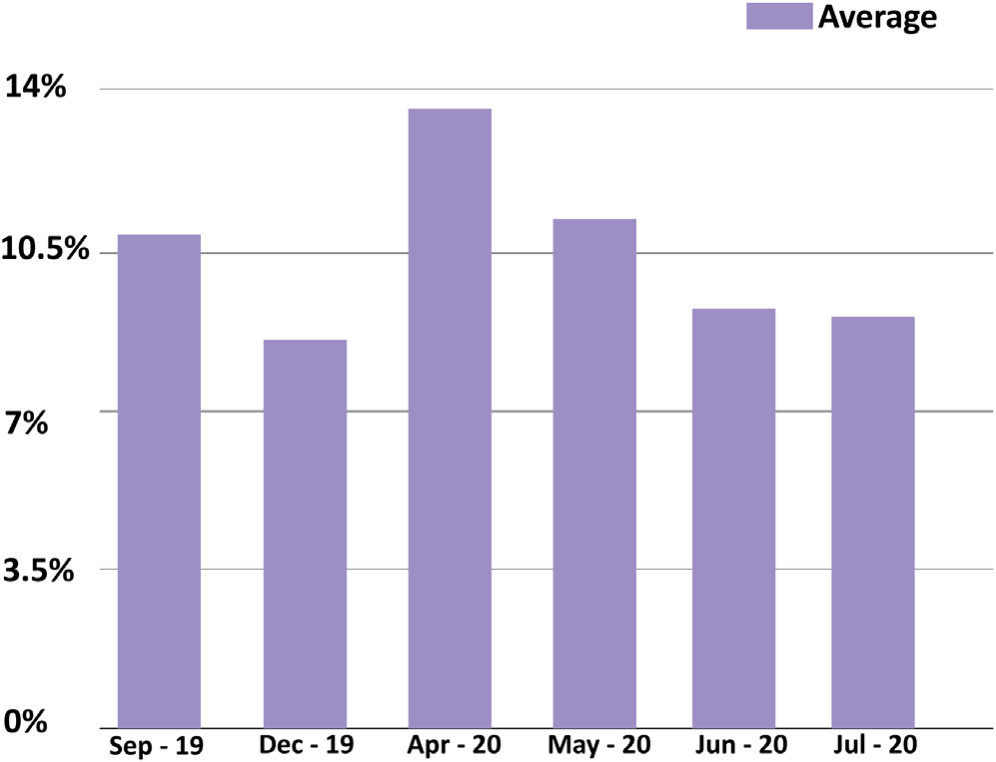
Representation of the average percentage of STAT tests exceeding the specified TAT along 6 months in the Clinical Chemistry, Hematology, and Parasitology

**FIGURE 2:**
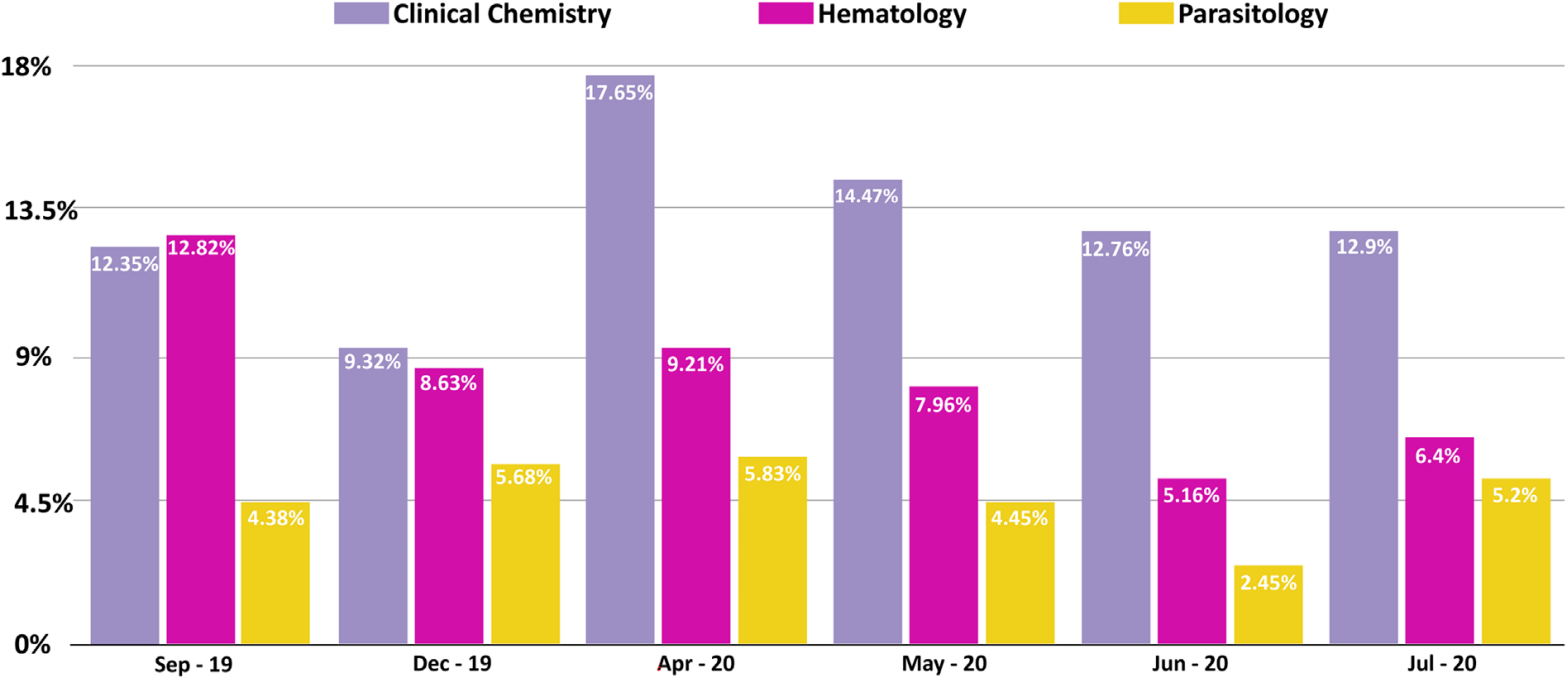
Representation of the percentage of STAT tests exceeding the specified TAT along 6 months in the Clinical Chemistry, Hematology, and Parasitology

To be able to assess staff's productivity in the Clinical Laboratory, the Turn Around Time (TAT) of Short Turn Around Time (STAT) tests exceeding 60 min of results release was monitored. Figures 1 and 2 represent the percentage of STAT tests exceeding the specified TAT along the period of 6 months in the three sections.

Turnaround time is known to be the difference in time between sample reception and result reporting.

The average TAT of STAT tests exceeding 60 min of results release decreased from 10.85% in September 2019 to 9.19% in July 2020. In the Clinical Chemistry section, the TAT of STAT tests exceeding 60 min of results release was 12.35% in September 2019. This TAT decreased to 9.32% in December 2019 due to the decrease in workload imposed by the October revolution in Beirut. In mid‐January 2020, the Clinical Laboratory started the installation of the core Laboratory, the period of installation was reflected by the increase in the TAT of STAT tests exceeding 60 min of results release to 17.65% in April 2020. This TAT decreased from 14.47% in May 2020 to 12.9% in July 2020. This decrease was due to the fact that the installation of the new pre‐analytical instrument required a long training period, staff confusion due to the additional tests which required supplementary controls and calibrators. In the Hematology section, the TAT of STAT tests exceeding 60 min of results release was 12.82% in September 2019. This relatively high TAT was due to the remoteness of the section from the receiving area in the laboratory. The TAT of STAT tests exceeding 60 min of results release decreased significantly from 9.21% in April 2020 to 6.4% in July 2020. This indicates that the installation of the core laboratory had a positive impact on the Hematology section particularly, by creating a common receiving area that allowed the Hematology section in the Clinical Laboratory to become more productive. The Parasitology section showed a moderately constant TAT of STAT tests exceeding 60 min of results release since no changes were enforced by the JCI standards implementation.

**FIGURE 3:**
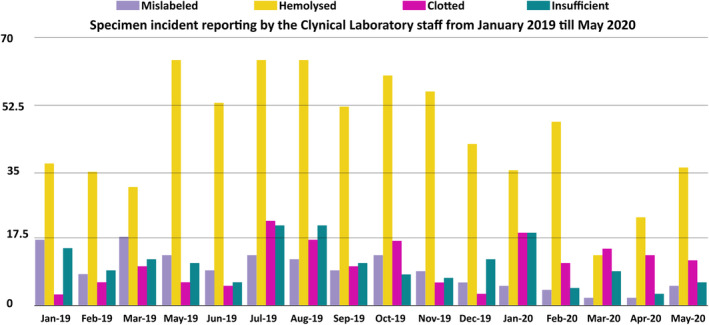
Representation of the number of rejected samples along a period of 16 months by the Nursing and Laboratory Phlebotomy teams

To measure staff's productivity from another aspect, analysis of the incident report forms was conducted. Figure 3 shows the distribution of rejected specimens collected by the Nursing and Clinical Laboratory teams from January 2019 till May 2020. Sample rejection criteria are essential in the process of result reporting. The pre‐analytical phase of result reporting includes but is not limited to specimen evaluation. Specimen rejection indicates quality assurance; this implies that samples are being checked for their consistency thus safeguarding quality of care. When samples are rejected, result reporting is delayed, thus staff productivity is altered. Thus, through the thorough analysis of the specimen incident reports filed by the receiving technologist at the Clinical Laboratory, the number of mislabeled specimens decreased from 17 samples in January 2019 to 5 mislabeled samples in May 2020. In addition, the number of insufficient samples decreased from 15 samples in January 2019 to 6 insufficient samples in May 2020. This decrease in specimen rejection represents the importance of JCI standards implementation in guaranteeing patient safety. This indicates that by implementing JCI standards specifically through continuous training of staff, decreases identification incidents and insufficiency thus enhancing a quality environment in result reporting. By improving the quality of samples sent to the Clinical Laboratory, the delay in result reporting is minimized thus the productivity increases.

In order to evaluate the staff's motivation along the process of JCI standards implementation, a questionnaire was distributed among the Clinical Laboratory and Blood Bank Technologists. The questionnaire was built to rely on the staff's experience, their awareness of JCI standards and how the management decisions are impacting their motivation toward their work duties. A two‐tailed chi‐square test of significance was conducted to assess the relationship between the qualitative variables. Since the sample size is small (less than 30 participants), and 80% of the expected values are less than 5, Fisher Exact test was selected to test our hypothesizes.

The relationship between staff experience and satisfaction level was assessed and represented in Table 1.

**TABLE 1:** Shows the staff satisfaction level according to their corresponding experience

		Staff experience				
		<1 year	<10 years	<25 years	>25 years	Total
Satisfaction	Minimal satisfaction	0	0	0	1	1
	Neutral	0	8	2	3	13
	Satisfied	0	1	0	7	8
	Extremely satisfied	1	1	0	2	4

*Note*: A significant relationship exists between staff experience and satisfaction level. *P* value = 0.031 which is less than 0.05, which indicates the significance of the relationship between the two studied variables; staff satisfaction and experience level.

Satisfaction and extreme satisfaction were relatively exclusive to experienced staff, meaning with more than 25 years of experience. Neutrality and minimal satisfaction were distributed among staff with 10 to 25 years of experience. However, the neutrality in satisfaction represented 50% of the population. Fisher exact test showed that our results are significant and H0 was rejected. This indicates that staff experience is dependent on satisfaction, since staff retention is built on satisfaction.

To study the impact of JCI standards implementation from another aspect, the relationship between staff's motivation during the process of standards implementation and staff experience level was monitored and represented in Table 2.

**TABLE 2:** Shows the staff's motivation during the process of JCI standards implementation with respect to years of experience

		Staff experience				
		<1 year	<10 years	<25 years	>25 years	Total
Motivation	Disagree	0	2	0	1	3
	Neutral	0	6	2	4	12
	Agree	0	2	0	8	10
	Strongly agree	1	0	0	0	1

*Note*: a significant relationship exists between staff experience and motivation level during the process of JCI standards implementation. *P* value = 0.034 which is less than 0.05, thus highlighting on the significant relationship between staff motivation and experience level.

Neutrality was predominant in the assessed correlation between staff experience and motivation during the process of JCI standards implementation. However, this neutrality was generally distributed among staff with less than 25 years of experience. Highly motivated staff are predominantly experienced with more than 25 years of experience. This high motivation during the process of JCI standards implementation among staff with more than 25 years of experience is due to the conformity with new managerial decisions and changes, which can also be linked to their satisfaction level. On the other side, the neutrality among staff with less than 25 years of experience is related to their resistance to change due to their relatively low satisfaction level. Fisher Exact test showed that a significant relationship exists between staff motivation during the process of JCI standards implementation and staff experience level, thus we reject H0.

Finally, Staff experience is dependent on satisfaction, since satisfaction is directly related to staff retention. Satisfied staff usually tends to accept managerial decisions which is reflected on their motivation toward improvement plans imposed by the management team. Thus, experienced staff tend to be more satisfied and yet more motivated. This study was able to prove that JCI standard implementation increase staff productivity through organizational development, quality improvement, and control. However, its impact on staff satisfaction and motivation was minimal specially in staff with less than 25 years of experience which is due to several reasons that are manageable through proper change management.

## 3 CONCLUSION

It is important to shed light on the fact that accreditation will not guarantee success and improvement in health care service delivery. On the contrary, accreditation is to be used as an evaluation method of the standards, policies, and procedures implemented at the HCO. While trying to implement accreditation standards at the HCO, the management's challenge is to ensure staff involvement to minimize resistance. Accreditation improves perceived quality through ensuring patient safety. Organizational changes imposed by quality improvement plans and accreditation standards impact staff's productivity through decreasing TAT of STAT tests, as well as minimizing recurrent specimen rejection due to training sessions, educational lectures, and continuous auditing of the proper application of policies and procedures. However, the organizational changes decrease staff motivation toward improvement. Therefore, to enhance staff motivation during the process of JCI standards implementation, a change management model will be developed in reference to the ADKAR change management model. To build the most appropriate and convenient model, a root‐cause analysis was made based on Ishikawa Diagram.

**FIGURE 4:**
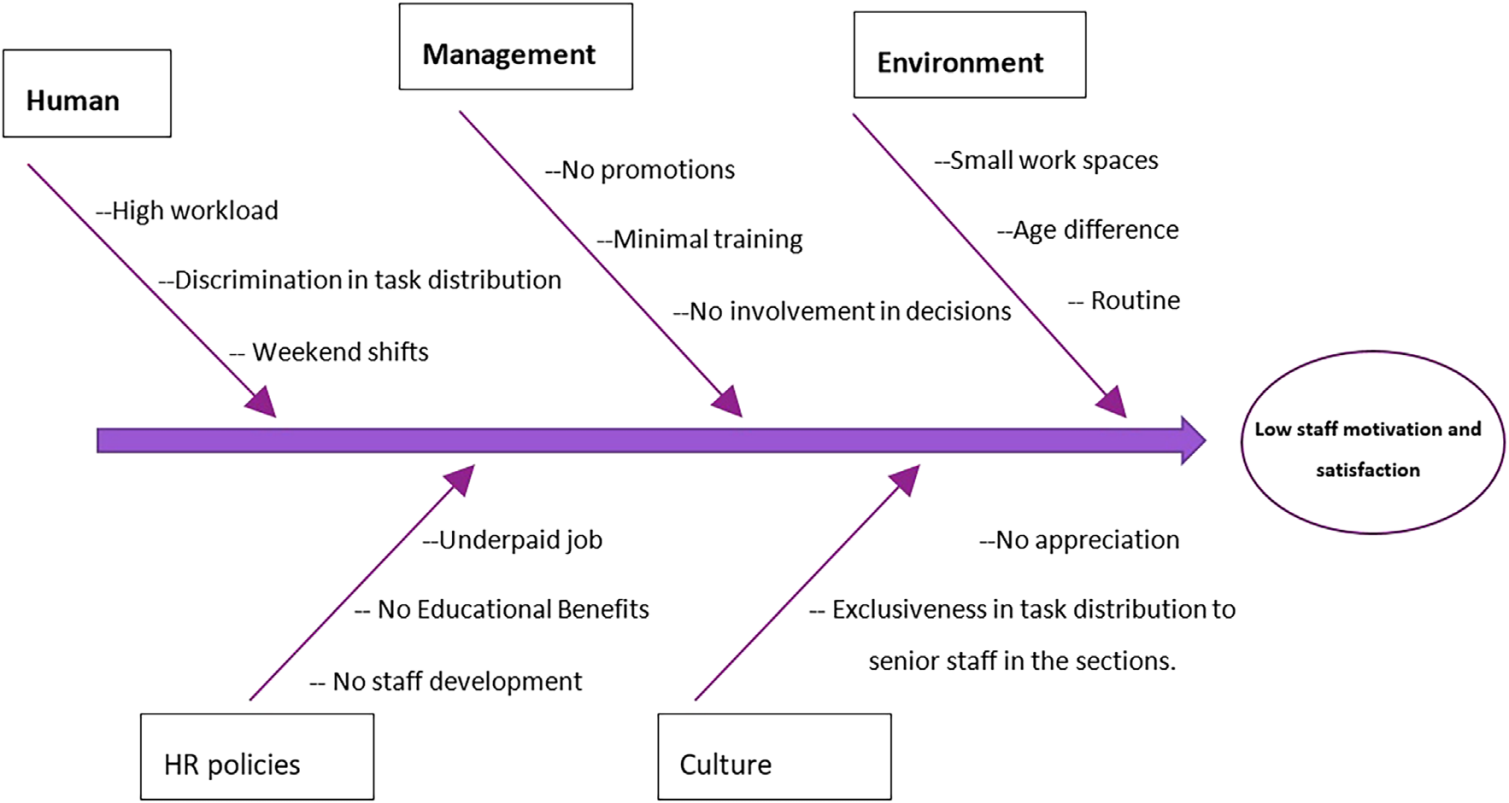
Root‐Cause analysis of low staff motivation and satisfaction

Figure 4 sheds the light on the different causes of staff demotivation and dissatisfaction. The causes were derived from the open‐ended questions of the distributed questionnaire and were grouped accordingly. By studying the causes of lower levels of satisfaction and motivation among staff with less than 25 years of experience, the management will be able to implement a change management model that will enhance and simplify the process of accreditation through decreasing resistance levels.

**FIGURE 5:**
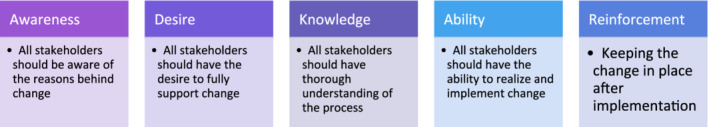
ADKAR change management model building blocks

ADKAR change management model was developed by Jeff Hiat as a potent tool for managers to sustain and manage successful change. ADKAR stands for 5 sequential building blocks crucial to stimulate successful change at the staff and management level.^10^ Figure 5 explains the building blocks of the ADKAR change management model. While implementing change, the employees are required to step out of their comfort zone. This is where the management roles lie in ensuring that all staff are conscious of the need to change. To create awareness of the urge to change, management bodies have to trigger all affected staff. This is can be achieved through the sharing of the uncomfortable truth and the existing gaps in the workflow. When the gaps are identified, stakeholders will understand the need to change. Understanding change is different from wanting the change to happen. Stakeholders should have the will to change, this is accomplished when the outcomes of change are reflected directly on the staff's benefits. Change cannot be implemented unless staff is knowledgeable about how the things will be done, which is completed through training. However, knowledge is not enough to have the job done if the staff does not have the ability to complete the task. Gaining the ability to complete the required tasks mandates hands‐on training, which is considered a way to avoid costly errors later on. Being knowledgeable and having the ability to perform a task does not create a long‐term habit. Reinforcement will be an ongoing process that will ensure that change is continuous. Management bodies should give permanent feedback on the process of change, and share the outcome with the stakeholders. Recognition is one way that guarantees the sustainability of change. This boosts staff motivation during the process of change, since it reflects on their important role in reaching the organization's desired goal. Monitoring the process of change benefits the management body as well, since it allows managers to interfere and fill the formed gaps before the system cracks.

The field of accreditation is under massive development, especially in healthcare organizations. Different accreditation bodies are available where most of them are patient centered. JCI accreditation is one of the accreditation bodies that require a lot of documentation and quality improvement to guarantee proper standard application. Change management is the most essential tool to ensure a successful process. This is achieved through the implementation of the ADKAR change management model will allow the organization to enhance staff motivation, which ensures staff retention and therefore increase productivity.

## CONFLICT OF INTEREST

The authors declare that they have no conflict of interest.

## AUTHOR CONTRIBUTIONS

Conceptualization: Rima Ramzi Abou Tarieh, Hanady Rafic Samaha.

Formal Analysis: Rima Ramzi Abou Tarieh, Hanady Rafic Samaha, Rania Nazir Naoufal.

Supervision: Hanady Rafic Samaha, Rania Nazir Naoufal, Ramez Zayyat.

Resources: Hanady Rafic Samaha, Rania Nazir Naoufa.

Validation: Hanady Rafic Samaha, Rania Nazir Naoufal.

Writing ‐ original draft: Rima Ramzi Abou Tarieh.

Writing‐ review and editing: Rima Ramzi Abou Tarieh, Hanady Rafic Samaha, Rania Nazir Naoufal.

All authors have read and approved the final version of the manuscript [CORRESPONDING AUTHOR or MANUSCRIPT GUARANTOR] had full access to all the data in this study and takes complete responsibility for the integrity of the data and the accuracy of the data analysis.

Rima Ramzi Abou Tarieh affirms that this manuscript is an honest, accurate, and transparent account of the study being reported; that no important aspects of the study have been omitted; and that any discrepancies from the study as planned (and, if relevant, registered) have been explained.

## TRANSPARENCY STATEMENT

The authors confirm this manuscript is an honest, accurate, and trans‐parent account of the study being reported; that no important aspects of the study have been omitted; and that any discrepancies from the study as planned (and, if relevant, registered) have been explained.

## ETHICS STATEMENT

The IRB/REC of University of Balamand/Saint Georges Hospital University Medical Center approved the use of SGHUMC data and under these terms the study was permitted.

## Data Availability

The authors confirm that the data supporting the findings of this study are available within the article [and/or] its supplementary materials.
